# Low‐Cost, High‐Pressure‐Synthesized Oxygen‐Entrapping Materials to Improve Treatment of Solid Tumors

**DOI:** 10.1002/advs.202205995

**Published:** 2023-02-02

**Authors:** Jianling Bi, Emily Witt, Vanessa A. Voltarelli, Vivian R. Feig, Veena Venkatachalam, Hannah Boyce, Megan McGovern, Wade R. Gutierrez, Jeffrey D. Rytlewski, Kate R. Bowman, Ashley C. Rhodes, Austin N. Cook, Benjamin N. Muller, Matthew G. Smith, Alexis Rebecca Ramos, Heena Panchal, Rebecca D. Dodd, Michael D. Henry, Adam Mailloux, Giovanni Traverso, Leo E. Otterbein, James D. Byrne

**Affiliations:** ^1^ Department of Radiation Oncology University of Iowa 200 Hawkins Drive Iowa City IA 52242 USA; ^2^ Department of Biomedical Engineering University of Iowa 200 Hawkins Drive Iowa City IA 52242 USA; ^3^ Holden Comprehensive Cancer Center University of Iowa Iowa City IA 52242 USA; ^4^ Department of Surgery Beth Israel Deaconess Medical Center Harvard Medical School 3 Blackfan Circle Boston MA 02215 USA; ^5^ Division of Gastroenterology Brigham and Women's Hospital Harvard Medical School 75 Francis St. Boston MA 02115 USA; ^6^ David H. Koch Institute for Integrative Cancer Research Massachusetts Institute of Technology 500 Main St Building 76 Cambridge MA 02142 USA; ^7^ Department of Mechanical Engineering Massachusetts Institute of Technology 77 Massachusetts Ave Cambridge MA 02139 USA; ^8^ Department of Systems Biology Harvard Medical School 75 Francis St. Boston MA 02115 USA; ^9^ Department of Radiation Oncology Brigham and Women's Hospital Harvard Medical School 75 Francis St. Boston MA 02115 USA; ^10^ Department of Chemical Engineering Massachusetts Institute of Technology 25 Ames St. Cambridge MA 02139 USA; ^11^ Medical Scientist Training Program University of Iowa Iowa City IA 52242 USA; ^12^ Department of Internal Medicine University of Iowa Iowa City IA 52242 USA; ^13^ Department of Microbiology and Immunology Carver College of Medicine University of Iowa Iowa City IA 52242 USA; ^14^ Department of Molecular Physiology and Biophysics Carver College of Medicine University of Iowa Iowa City IA 52242 USA

**Keywords:** gas‐entrapping materials, malignant peripheral nerve sheath tumors, radiation therapy, tumor hypoxia

## Abstract

Tumor hypoxia drives resistance to many cancer therapies, including radiotherapy and chemotherapy. Methods that increase tumor oxygen pressures, such as hyperbaric oxygen therapy and microbubble infusion, are utilized to improve the responses to current standard‐of‐care therapies. However, key obstacles remain, in particular delivery of oxygen at the appropriate dose and with optimal pharmacokinetics. Toward overcoming these hurdles, gas‐entrapping materials (GeMs) that are capable of tunable oxygen release are formulated. It is shown that injection or implantation of these materials into tumors can mitigate tumor hypoxia by delivering oxygen locally and that these GeMs enhance responsiveness to radiation and chemotherapy in multiple tumor types. This paper also demonstrates, by comparing an oxygen (O_2_)‐GeM to a sham GeM, that the former generates an antitumorigenic and immunogenic tumor microenvironment in malignant peripheral nerve sheath tumors. Collectively the results indicate that the use of O_2_‐GeMs is promising as an adjunctive strategy for the treatment of solid tumors.

## Graphical abstract

A novel class of oxygen‐releasing materials is developed using molecular gastronomy techniques. Injection or implantation of these materials into tumors prolongs oxygen delivery to tumors and results in tumor hyperoxia, and in combination with radiation or chemotherapy this treatment significantly inhibits tumor growth and promotes survival.

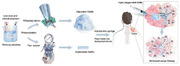

## 1 Introduction

Hypoxia is a condition that drives lack of responses to a wide array of cancer therapies.^[^ ^1^ ^]^ It arises because the rapid growth of tumors can lead to aberrant vascular proliferation and create heterogenous areas of low oxygen pressure and hypoxic stress. This hypoxic stress can in turn force a more aggressive phenotype characterized by increased invasiveness and metastasis, and when chronic, such stress can further lead to the development of resistance to chemotherapy, radiation, or immunotherapy. The hypoxic environment contributes to nutrient deficits and acidosis, and this accelerates stress in the tumor microenvironment, ultimately, impairing the response to therapy. Because all cancer types are characterized by hypoxia in and surrounding the tumor, this condition has long been recognized as a target for therapies; however, altering the tumor microenvironment has proven technically challenging.^[^ ^2^ ^]^

Several methods that increase oxygen levels in tumors (e.g., the application of hyperbaric oxygen therapy or oxygen‐filled microbubbles) or exploit low tumor oxygen levels (e.g., implanting *C. novyi*, administering tirapazamine) have been shown to improve responses to current standard‐of‐care therapies.^[^ ^3^ ^]^ Of these, only hyperbaric oxygen therapy has been tested clinically in subjects with cancer undergoing radiation therapy. However, the effectiveness of this approach is limited by the lack of access to equipment needed as well as by potential toxicity of oxygen to normal tissue.^[^ ^3a,b^ ^]^ Other methods, including injection of oxygen‐filled microbubbles, have proven effective in tumor‐bearing preclinical mouse models.^[^ ^3d,e^ ^]^ The key challenges in most of these approaches are achieving optimal intratumoral oxygen pressures and pharmacokinetics, safety, and ideal treatment logistics.

Altering oxygen levels in the tumor not only has the potential to enhance the response to anticancer therapy, but also represents a novel therapeutic strategy in cases of tumors that are inoperable or patients who are poor surgical candidates.^[^ ^4^ ^]^ One such scenario is malignant peripheral nerve sheath tumors (MPNSTs), which wrap around peripheral nerves; thus their surgical resection could damage the nerve, and therefore result in paralysis, severe morbidity, or even death.^[^ ^5^ ^]^ We hypothesized that biomaterials that enable more controlled delivery of oxygen would more effectively mitigate tumor hypoxia and result in improved management of inoperable or treatment‐resistant tumors when combined with current standard‐of‐care therapies. Toward developing such materials, we looked to molecular gastronomy. This area of the culinary arts focuses on the physicochemical transformation of foods, and the techniques used are directly translatable to the field of materials science for gas‐entrapment and delivery.

Herein, we report on our engineering of novel, low‐cost, high‐pressure‐synthesized materials, using Food and Drug Administration generally regarded as safe (GRAS) components, for intratumoral delivery of oxygen. GRAS materials leverage biocompatible and scalable natural polymers for tunable oxygen release. We show that their unique properties enable facile administration via direct injection. We also show that the creation of an oxygen‐rich tumor microenvironment synergizes with radiation and chemotherapy, profoundly improving their efficacy in the treatment of solid tumors in mice. Finally, we show that the tumor microenvironment changes uniquely following O_2_‐GeM exposure, suggesting that this approach can change the immune milieu of tumors and enhance the effectiveness of immunotherapies.

## 2 Results

### 2.1 Development of Injectable O_2_‐GeMs to Overcome Tumor Hypoxia

**Figure 1:**
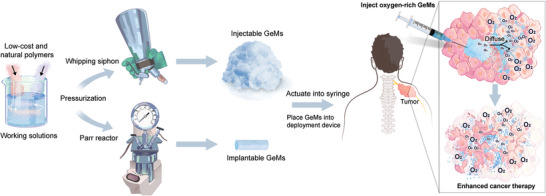
Schematic diagram illustrating how O_2_‐GeMs are fabricated and incorporated into clinically relevant therapies for treatment‐refractory tumors, such as MPNSTs.

**Figure 2:**
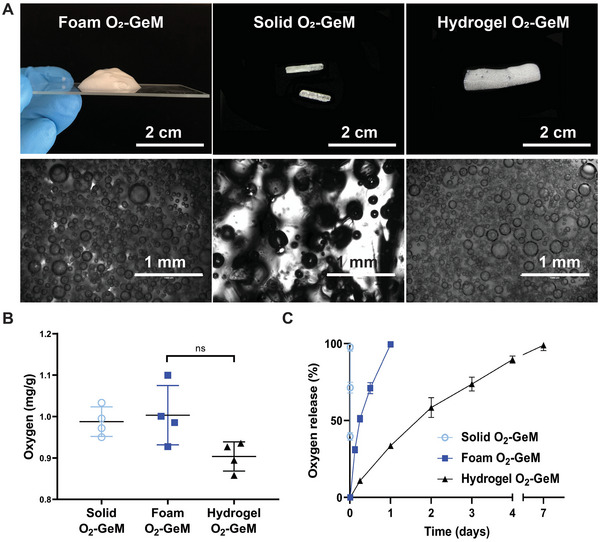
O_2_‐GeMs composed of GRAS materials. A) Images of three O_2_‐GeM formulations (a foam, a solid, and a hydrogel) viewed macroscopically (upper panels) and microscopically in clinically relevant dosage forms (lower panels). B) Concentration of oxygen in each formulation. C) Kinetics of oxygen release from each formulation. Data are shown as means and standard deviations (*n* = 4 per arm). *P* values were determined by one‐way ANOVA with multiple comparisons. NS, not significant.

**Figure** **1** depicts the clinical workflow for generating O_2_‐GeMs using low‐cost materials and high‐pressure vessels and then introducing the materials into a tumor to mitigate hypoxia and improve the response to standard‐of‐care cancer treatments. High‐pressure vessels commonly used in the culinary arts were employed to generate the following three O_2_‐GeM formulations: a foam, a solid, and a hydrogel (**Figure** **2**A). The GeMs are composed of GRAS components, including xanthan gum and sodium alginate, which are commonly used as inactive ingredients in manufacturing pharmaceuticals (e.g., atorvastatin and ketoconazole).^[^ ^6^ ^]^

To fabricate foam and hydrogel O_2_‐GeMs, we modified a whipping siphon for pressurization with oxygen gas (Figure S1, Supporting Information). The use of a whipping siphon made it possible to evaluate different foaming agents with tunable material properties. To fabricate solid O_2_‐GeM, we used a Parr reactor (Figure S2, Supporting Information). This physically entrapped pressurized oxygen in a natural polymer matrix, as is done to manufacture the candy Pop Rocks.^[^ ^7^ ^]^ A broad array of natural polymers was tested for their gas entrapment capabilities, and those of multiple natural carbohydrate‐based materials were found to be qualitatively similar to the candy Pop Rocks as defined by the pop rating in the literature on gasified candy (Figure S3, Supporting Information).^[^ ^7^ ^]^ An autosampler with cylindrical tubing was attached to the Parr reactor, enabling generation of cylindrical solid O_2_‐GeM made of sucrose, lactose, corn syrup, and water. This is a clinically relevant deliverable form whose size and shape (5 × 0.8 mm) are similar to those of brachytherapy spacers and gold fiducials used for radiographic localization of tumors and organs (Figures S2 and S4, Supporting Information). Gas chromatography with a thermal conductivity detector was used to quantify the amount of oxygen entrapped in the foam, solid, and hydrogel GeMs. The oxygen concentration was approximately the same in all three (Figure 2B), and each material entrapped twice as much oxygen as previously studied materials (**Table** **1**).^[^ ^8^, ^24^, ^33^ ^]^ In addition, the density of the foam O_2_‐GeMs was significantly lower than those of hydrogel and solid O_2_‐GeMs (Figure S5, Supporting Information).

**Table 1:** Summary of published oxygen‐releasing materials, including the O_2_‐GeMs, and their oxygen content

Material/form	Material [O_2_] [mg g^−1^]	O_2_ partial pressure intratumorally [mmHg]	Refs.
O_2_‐GeMs	1.0	>200	Not applicable
Perfluorotributylamine nanoparticles	0.016	x	^[^ ^24^ ^]^
Polymerized human hemoglobin	0.0069	30	^[^ ^25^ ^]^
Perfluorochemical nanoemulsions	0.43	x	^[^ ^26^ ^]^
O_2_ microbubbles w/ultrasound	0.097	x	^[^ ^3c^ ^]^
Water‐splitting nanoparticles	0.0015	x	^[^ ^27^ ^]^
O_2_ carrying hydrogels w/radiotherapy	0.095	x	^[^ ^28^ ^]^
O_2_‐generating hydrogels	0.004	12	^[^ ^29^ ^]^
pH sensitive O_2_ nanobubbles	0.0049	27	^[^ ^30^ ^]^
RBC O_2_ delivering microvehicles	0.0013	x	^[^ ^31^ ^]^
O_2_ encapsulating polydopamine nanoparticles	0.0069	19.7	^[^ ^32^ ^]^
Cyanobacteria O_2_ pump	0.0084	x	^[^ ^33^ ^]^

### 2.2 Assessment of Tunable Properties of GeMs

Tunable release of oxygen over time scales relevant to cancer therapy is critical for maximizing the benefit of mitigating tumor hypoxia. For example, GeMs that enable release of oxygen prior to and during irradiation would be beneficial for maximal generation of reactive oxygen species and deoxyribonucleic acid (DNA) fixation.^[^ ^9^ ^]^ We tested several GeM formulations in vitro and found that they led to oxygen release on different timeframes: the hydrogel O_2_‐GeM (ionically crosslinked matrix) released oxygen over the course of 1 week; the foam O_2_‐GeM (non‐crosslinked matrix) released oxygen over the course of hours; and the solid O_2_‐GeM (hygroscopic matrix that dissolves rapidly) released oxygen over the course of minutes (Figure 2C). In the case of both foams and hydrogels, oxygen release can be further controlled by changing the composition of the materials (the percentage of xanthan gum in foams and that of alginate in the hydrogels) (Figure S6, Supporting Information), and in the case of foams it can be further controlled by the O_2_ percentage (Figure S7, Supporting Information). For the solid GeM, we tested the effects of adding single or multiple layers of a commercially available biocompatible and non‐cytotoxic fluorinated polymer, FluoroPel 800, and found that dissolution of the rapidly dissolvable matrix was prolonged as a function of the number of layers (Figure S8, Supporting Information). The biocompatibility of the GeMs and FluoroPel in MyC‐CaP cells was confirmed using an alamarBlue cell viability assay (Figure S9, Supporting Information).

We next evaluated the mechanical properties of the candidate foam and hydrogel O_2_‐GeMs to determine whether they would be appropriate to use in subsequent analyses. The focus shifted to these materials because of the timeframe for oxygen release and greater range of control, which are best suited for clinical application. Foam O_2_‐GeM with varying concentrations of xanthan gum was subjected to rheological assessment to determine the behavior under flow conditions. The foam O_2_‐GeM exhibited shear‐thinning without fracture at high shear rates, and thus can easily be administered through a hypodermic needle or catheter. The advantages of the foam formulations are several fold: following injection and shear they self‐heal to a solid‐like state, forming robust depots, and this can contribute to intratumoral persistence and prolonged oxygen delivery (Figure S10, Supporting Information); both the viscosity and storage moduli increase as a function of polymer concentration and thus enable oxygen retention and prolonged oxygen release. Foam and hydrogel O_2_‐GeMs were then compared for material stiffness, and the hydrogel GeM demonstrates a higher degree of stiffness resulting (Figure S10E, Supporting Information) in prolonged oxygen release compared to the foam GeM. The matrix of the solid O_2_‐GeMs was subjected to compressive testing and was found to have an average fracture strength of 0.57 MPa indicating pressurized gas could be retained within the matrix.

### 2.3 Effects of GeM‐Mediated Delivery and Diffusion of Oxygen in Tumor Tissue Surrogates and Whole Tumors

**Figure 3:**
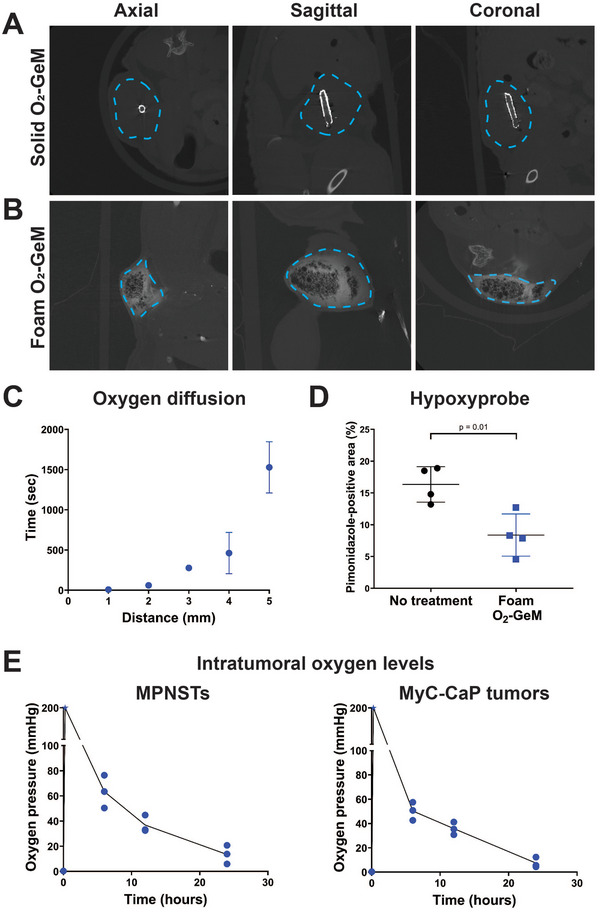
Intratumoral administration of O_2_‐GeMs and the impact on oxygen pressure in mouse tumors. A,B) Representative images from CT scans of subcutaneous syngeneic MPNSTs with intratumorally (A) implanted solid O_2_‐GeM and (B) injected foam O_2_‐GeM. Each tumor is outlined by a blue dotted line. The solid O_2_‐GeM was coated with a high‐Z material; the foam O_2_‐GeM incorporated the iodinated contrast compound Iohexol at a 10% volume/volume ratio. C) Oxygen diffusion within a tumor tissue surrogate (2 wt% agarose gel). The times recorded are those needed to reach 20 mmHg above baseline at oxygen probes implanted in the surrogate at the indicated distances (*n* = 4 per mm of distance). D) Quantitation of tumor oxygen pressure, using the hypoxia marker pimonidazole, in subcutaneous MPNSTs one day after intratumoral injection of foam O_2_‐GeMs. *P* values were determined by unpaired *t*‐test (*n* = 4 mice per arm). E) Changes in oxygen pressure in subcutaneous syngeneic MPNST and subcutaneous syngeneic prostate tumors injected with foam O_2_‐GeM (*n* = 3 per time point). For both tumor types, baseline tumor oxygen pressures were between 0.4 and 0.5 mmHg.

To test the feasibility of clinical translation of O_2_‐GeMs to treating solid tumors, we evaluated the efficacy of implanting solid O_2_‐GeMs and injecting foam O_2_‐GeMs (**Figure** **3**A,B). For ease of visualization of the material, solid O_2_‐GeMs (5 × 0.8 mm, length × diameter) were coated with a high‐Z material and then implanted directly into a subcutaneous MPNST. The solid O_2_‐GeM was shown to be well‐centered in each tumor (Figure 3A). In a separate group of MPNST‐bearing mice, tumors were injected with 50 µL of a foam O_2_‐GeM that contained 10% volume/volume of the iodinated contrast agent, Iohexol, and depot formation within the tumor was visualized (Figure 3B). Notably, the entrapped oxygen was easily identified as hypodensities within the tumor bed. The ease of positioning of the solid and foam O_2_‐GeMs in tumors is consistent with successful translation to the clinical setting.

Next, we measured the diffusion of oxygen from the foam O_2_‐GeM through a tumor tissue surrogate (2 wt% agarose) using a single oxygen probe implanted at several distances from the site of the O_2_‐GeM. The time to reach an oxygen concentration 20 mmHg above baseline increased with the distance from the GeM (Figure 3C). This finding was supported by staining of MPNST with the hypoxia indicator pimonidazole (Hypoxyprobe). By one day after injection with foam O_2_‐GeM (50 µL), pimonidazole staining in the tumors was significantly lower than in no treatment controls (Figure 3D). Moreover, the kinetics of change in tumor oxygen pressures were evaluated after two types of flank tumors, syngeneic MPNST and syngeneic prostate (MyC‐CaP), were injected with foam O_2_‐GeMs (Figure 3E). Measurements were made using oxygen probes that were intratumorally implanted at designated times after foam injection (baseline (no foam), 15 min, 6 h, 12 h, and 24 h) (Figure 3E). Tumor oxygen pressures were found to be above the limit of quantitation (>200 mmHg) immediately after placement, and they decreased over the course of 24 h, at which point levels were still at or above the oxygen partial pressure of 15 mmHg, a threshold that distinguishes between pathologic tumor hypoxia and the physiologic hypoxia that characterizes the liver and bone marrow.^[^ ^10^ ^]^ In addition, the 24‐h levels were found to be well above baseline tumor oxygen pressures (≤2.5 mmHg in both tumor types). An important consideration for each GeM is its degradation time. The solid O_2_‐GeMs were degraded within minutes, whereas the foam O_2_‐GeMs may release gas over the course of a day and may degrade over a period of months.

### 2.4 Effects of O_2_‐GeMs on the Effectiveness of Cancer Therapy in Solid Tumors

**Figure 4:**
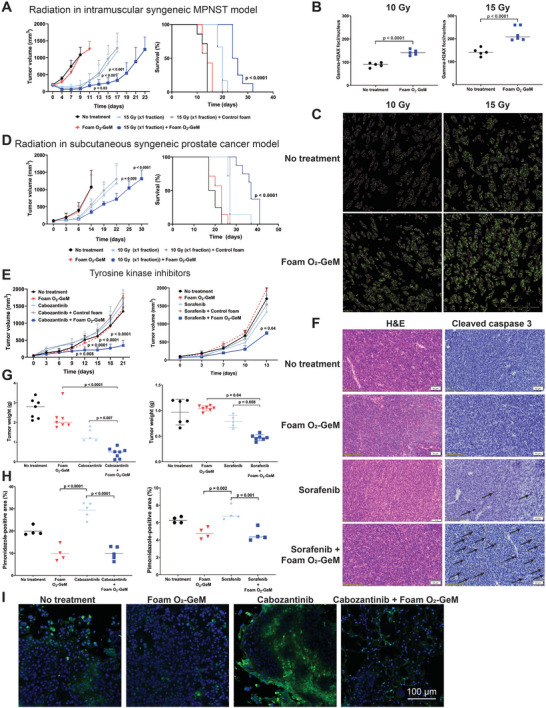
Therapeutic effect of the concomitant administration of foam O_2_‐GeM and radiation or chemotherapy in tumor‐bearing mice. A) Effects on tumor volume and animal survival when intramuscular syngeneic MPNSTs were injected with foam O_2_‐GeM up to 30 min before irradiation (15 Gy). Controls included nongasified foam + 15 Gy, 15 Gy, foam O_2_‐GeM, and no treatment. Data are shown as means and standard deviations (*n* = 7 per arm). *P* values were determined by one‐way ANOVA with multiple comparisons. B) Quantification of *γ*‐H2AX foci/nucleus in MPNST 1 h after implantation of foam O_2_‐GeMs + radiation (15 Gy and 10 Gy in separate arms). *P* values were determined by unpaired *t* test. C) Representative images of *γ*‐H2AX foci in nuclei, as identified using Cell Profiler. D) Effects on tumor volume and animal survival when subcutaneous syngeneic prostate tumors were injected with foam O_2_‐GeM up to 30 min before irradiation (10 Gy). Controls included nongasified foam + 10 Gy, 10 Gy, foam O_2_‐GeM, and no treatment. Data are means and standard deviations (*n* = 7 per arm). *P* values were determined by one‐way ANOVA with multiple comparisons. E) Effects on tumor volume when intramuscular syngeneic MPNSTs were injected with foam O_2_‐GeMs twice weekly and orally administered tyrosine kinase inhibitor (TKI) was administered daily. (Left panel) Application of the TKI cabozantinib. (Right panel) Application of the TKI sorafenib. Controls included nongasified foam + TKI, TKI, foam O_2_‐GeM, and no treatment. Data are means and standard deviations (*n* = 7 per arm). *P* values were determined by one‐way ANOVA with multiple comparisons. F) H&E and cleaved caspase 3 staining of MPNSTs following treatment with foam GeM and/or sorafenib. G) Weights of tumors collected at 21 days following treatment with foam GeM and/or TKI inhibitor (left panel, cabozantinib; right panel, sorafenib). Data are means and standard deviations (*n* = 7 per arm). *P* values were determined by one‐way ANOVA with multiple comparisons. H) Quantitation of tumor oxygen pressure, using the hypoxia marker pimonidazole, in subcutaneous MPNSTs and prostate tumors (MyC‐CaP) one day after intratumoral injection of foam O_2_‐GeM. Data are means and standard deviations (*n* = 4–5 per arm). *P* values were determined by one‐way ANOVA with multiple comparisons. I) Representative histologic images of pimonidazole and DAPI staining in subcutaneous MyC‐CaP tumors 6 h after treatment. Levels of pimidazole staining were generally lower in the samples treated with foam O_2_‐GeM than that in controls. Data are individual values (*n* = 4–5 slides per arm).

To evaluate the utility of O_2_‐GeMs as an adjunctive strategy with radiation and chemotherapy, we performed efficacy studies in two syngeneic tumor models—intramuscular (flank) syngeneic MPNST and subcutaneous syngeneic prostate cancer (MyC‐CaP) models. Based upon their diffusion, rapid release of oxygen, and ease of injection, the foam O_2_‐GeM was chosen for this analysis. To ensure that the environment in and around the tumor was oxygen‐rich, we injected tumors with 50 µL of the foam O_2_‐GeM and peri‐tumoral sites with 100 µL of the same formulation. In each tumor model, the foam O_2_‐GeM was administered 30 min prior to irradiation. The median MPNST size at the time of GeM injection was ≈160 mm^3^. A small animal radiation research platform (SARRP) was used, enabling localization of the tumors by CT guidance. For studies involving the MPNST‐bearing mice, the tumors were irradiated with 15 gray (Gy) (×1 fraction), 10 Gy (×2 fractions), or 8 Gy (×3 fractions), each of which is equivalent to a biological equivalent dose (BED) used for preoperative treatment of sarcoma tumors in humans, assuming an alpha–beta of 4.^[^ ^11^ ^]^ Foam O_2_‐GeM + radiation (15 Gy) inhibited tumor growth and extended the life span of mice with MPNSTs from a median time of 13 days (no treatment) to 25 days (foam O_2_‐GeM + radiation) (**Figure** **4** **A**); treatment at the same BED but different fractionation schemes resulted in outcomes similar to those for the 15 Gy treatment (Figure S11, Supporting Information). The data in Figure 4A,D,E are also represented as the ratio of tumor volume over time to initial tumor volume (*V*_t_/*V*_0_) in Figure S12 of the Supporting Information. Histological evaluation of *γ*‐H2AX foci, a marker of double strand DNA breaks, in tumors 1 h after a single radiation treatment (15 and 10 Gy) revealed a significantly larger number of *γ*‐H2AX foci/nucleus for the O_2_‐GeM + radiation versus radiation only control sample (Figure 4B,C). When the same injection volumes were used in the MyC‐CaP model, the median tumor size was ≈100 mm^3^. The tumors of prostate tumor‐bearing mice were irradiated with a single dose of 10 Gy, also using an SARRP. Tumor growth in the foam O_2_‐GeM + radiation treated mice was significantly slower than in their radiation‐only treated and no treatment control counterparts and extended the life span from a median of 20 days (no treatment) to 37 days (foam O_2_‐GeM + radiation) (Figure 4D).

The O_2_‐GeMs were then tested in combination with chemotherapeutic agents known to be more effective clinically at higher oxygen levels. Two tyrosine kinase inhibitors, cabozantinib and sorafenib, were previously found to be effective under normoxic conditions.^[^ ^12^ ^]^ Cabozantinib has been used in clinical trials for prostate cancer and sorafenib in the management of metastatic MPNST.^[^ ^13^ ^]^ We tested the in vitro efficacy of these agents at two oxygen levels in MPNST and MyC‐CaP cells and found that their efficacy was higher at normoxia than severe hypoxia (Figure S13, Supporting Information). Efficacy studies were also performed in the intramuscular syngeneic MPNST and the syngeneic prostate cancer mouse models to determine the effectiveness of the O_2_‐GeM as a combination strategy with the tyrosine kinase inhibitors (Figures 4E–I). Mice bearing prostate tumors were given daily doses of cabozantinib (60 mg kg^−1^) and twice weekly intratumoral injections of the foam O_2_‐GeM for a total of 21 days. The foam O_2_‐GeM + cabozantinib treatment significantly inhibited tumor growth relative to the cabozantinib treatment alone (Figure 4E (left), Figure 4G (left)); thus, cabozantinib is likely subtherapeutic as a single agent. Furthermore, multiple trials of cabozantinib in metastatic castrate‐resistant prostate cancer patients have shown no survival or pain palliation benefit.^[^ ^14^ ^]^ Mice bearing intramuscular MPNSTs were given daily doses of sorafenib (30 mg kg^−1^) and twice weekly intratumoral injections of foam O_2_‐GeM for a total of 13 days. The foam O_2_‐GeM + sorafenib treatment significantly inhibited tumor growth relative to the sorafenib treatment alone mice; Figure 4E (right), Figure 4G (left)). Histologic evaluation of cleaved caspase 3, a marker of apoptosis, revealed higher levels of staining in the tumors of mice treated with foam O_2_‐GeM + sorafenib than in their sorafenib, foam O_2_‐GeM, and no treatment control counterparts (Figure 4F). Figure 4H shows quantification of the hypoxia (pimonidazole staining) in histologic sections of tumor tissue from mice treated with these tyrosine kinase inhibitors and the foam O_2_‐GeM. The tyrosine kinase inhibitors were found to induce a greater degree of hypoxia in tumors than in controls. Addition of the foam O_2_‐GeM reversed this hypoxia, creating a less hypoxic environment. Figure 4I shows representative histologic pimonidazole staining and Figure 4H (right) shows its quantification; Figure S14 of the Supporting Information shows all other histology images used in the quantification for Figure 4H.

### 2.5 Effects of O_2_‐GeMs on the Immune Composition of Solid Tumors

**Figure 5:**
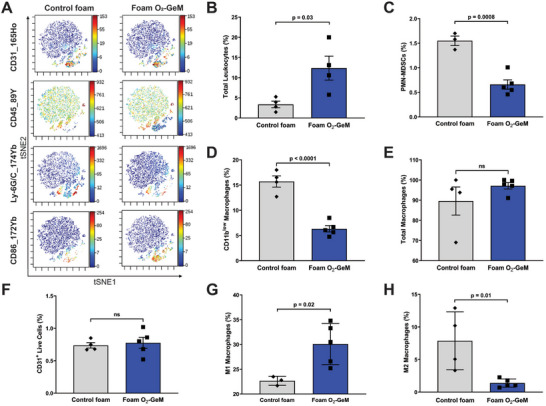
CyTOF analysis of intramuscular MPNST tumors following repeated treatment with the foam O_2_‐GeM. A) Histograms of cells stained for the indicated markers 6 h after receiving the final dose of the foam O_2_‐GeM. B–G) Quantification of the contribution of the indicated cell type to the tumor (% composition), in treated and control tumors. Data are shown as mean and standard deviations (*n* = 4–5 per group). *P* values were determined by unpaired *t*‐test.

To understand the influence of O_2_‐GeMs on the tumor immune cell landscape, we performed cytometry by time of flight (CyTOF) experiments on MPNSTs treated with the foam O_2_‐GeM and compared the results to those for control foam solution (no gas). **Figure** **5** demonstrates a significant difference in the immune‐cell profiles of tumors treated with these agents, as shown by both t‐distributed stochastic neighbor embedding (tSNE) plots (Figure 5A) and quantification of the cellular composition of the tumors (Figure 5B–H). Leukocyte infiltration was significantly more prominent, and the numbers of immunosuppressive cells (i.e., polymorphonuclear‐myeloid‐derived suppressor cells (PMN‐MDSCs) and CD11^low^ macrophages) were significantly lower in tumors treated with the foam O_2_‐GeM than in the controls. Furthermore, the percentage of M1 (antitumorigenic) macrophages was significantly greater and M2 (protumorigenic) macrophages significantly lower, in tumors treated with the foam O_2_‐GeM. Of note, the proportion of endothelial cells (CD31^+^) did not differ between groups.

**Figure 6:**
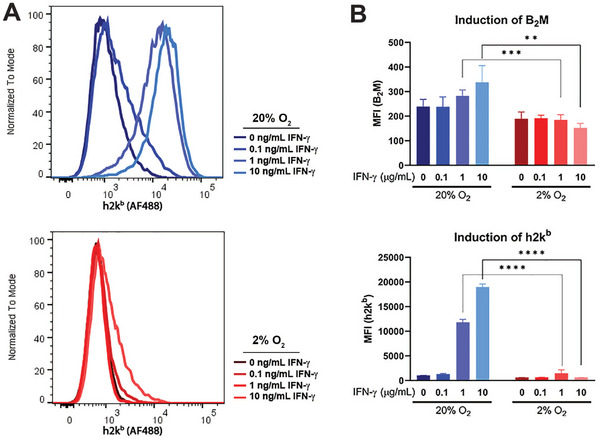
In vitro assessment of levels of major histocompatibility complex (MHC) class 1 (h2k^b^) on the surface of MPNST cells after exposure to the stimulatory molecule, interferon‐gamma (IFN‐*γ*), at 20% (blue, normoxia) versus 2% oxygen (red, hypoxia). A) Flow histograms for MPNST cells treated with varying doses of IFN‐*γ* under normoxia and hypoxia. B) Quantification of mean fluorescence intensity (MFI) for the MHC class I markers B_2_M and h2k^b^ Data are shown as mean and standard deviations (*n* = 5 per group). *P* values were determined by unpaired *t*‐test.

Elevated tumor oxygen pressure also plays an important role in the presentation of tumor‐cell antigens because it affects the expression of class I major histocompatibility complexes (MHCs) on the tumor cell surface.^[^ ^15^ ^]^ In vitro assessment of cell–surface murine MHC class I (h2k^b^) in MPNST cells revealed that levels were low under baseline conditions regardless of oxygen pressure, but that surface levels of h2k^b^ and beta‐2‐microglobulin (B_2_M) were elevated by stimulation with interferon gamma (IFN‐*γ*) when oxygen pressure was increased (**Figure** **6**). This raises the possibility that O_2_‐GeMs may convert MPNSTs from immunologically “cold” to “hot.”

## 3 Discussion

Tumor hypoxia represents a formidable challenge for effective cancer control. The clinical translation and uptake of technologies that address this problem has remained elusive. We decided to take an approach inspired by the culinary arts to deliver oxygen locally to tumors, using pressurized vessels to generate GeMs. These GeMs entrap large volumes of gas and can be easily delivered through hypodermic needles or catheter. Moreover, these GeMs can entrap a variety of gases.^[^ ^16^ ^]^

Here, we show that administering oxygen through a GeM approach increases intratumoral oxygen levels. Our preclinical results demonstrate that in two distinct animal models of cancer, the delivery of oxygen through these materials improved responses to radiation and chemotherapy. It is possible that multiple mechanisms can contribute to the demonstrated anticancer benefit of these materials. These include an elevation of reactive oxygen species levels, enhancement of drug delivery, and modification of the metabolic capacity of immune cells. We also show that these materials can change the immune landscape of tumors and have the potential to enhance tumor response to immunotherapies. These studies showcase the potential benefit of mitigating tumor hypoxia through simple strategies.^[^ ^2^ ^]^

Strategies to mitigate tumor hypoxia gained traction recently as the influence of low oxygen partial pressures on tumor growth has become clearer. Prior research showed that tumor hypoxia reduces the efficacy of certain chemotherapies over twofold and that of radiation over tenfold.^[^ ^17^ ^]^ Tumors frequently exhibit acute or chronic hypoxia, and both forms of hypoxia play a major role in dampening the response to cancer treatment.^[^ ^9b^ ^]^ Patients would benefit from an intervention that modulates oxygen partial pressures.^[^ ^18^ ^]^ The delivery of oxygen by a tunable matrix could profoundly influence the effectiveness of current cancer treatment by mitigating acute and chronic hypoxia. O_2_‐GeMs offer the possibility to 1) tune oxygen delivery, 2) achieve more dynamic intratumoral oxygen kinetics than has been possible to date, and 3) achieve maximum oxygen partial pressure. They also represent safe and biocompatible materials. They will likely be effective as adjuncts with other standard‐of‐care therapies.

Although we achieved significant tumor growth inhibition using an O_2_‐GeM in multiple tumor‐bearing models, several avenues of further research are apparent from this study. For example, it is possible that the efficacy of these materials can be further improved by concomitant administration of drugs (including immunotherapeutic agents) and/or other gases. Because the foam and hydrogel O_2_‐GeMs are composed of natural, biocompatible polymers that are not rapidly degraded, it may be beneficial to develop resorbable materials that are more rapidly degraded at the intratumoral injection site. A weakness of this study is that it did not investigate whether continuous O_2_‐GeM administration and/or combination therapy would be needed to eliminate the tumor burden. The fact that tumor growth was slowed rather than completely arrested by the tested regimens suggests that further treatments may be required. New approaches to be taken would include expanding the use of pressurized vessels to provide additional opportunities for optimization. For example, new materials and higher pressures could be tested to improve gas retention and delivery. These adjustments might reduce the volume required for delivering appropriate doses. Methods to enhance oxygen diffusion, such as local heating, might further mitigate hypoxia. It will also be critical to confirm that oxygen is not toxic to peri‐tumoral normal tissue. Finally, we will take advantage of our close partnership with interventionalists to facilitate effective placement of O_2_‐GeMs in patients, especially in the cases of deeper tumors.^[^ ^19^ ^]^

Translation of the system described here to human use should be rapid because it relies on simple GRAS components. Testing of these materials in large animal models as a next step would help facilitate rapid translation by testing in species that more closely approximate the human condition. Of note, we have tested a carbon monoxide‐GeM in the foam formulation and found that this system is amenable to systemic delivery of gases in both small and large animals.^[^ ^16^ ^]^ Another report has demonstrated that oxygen delivery through the gastrointestinal tract is an effective modality that may benefit patients with lung disease.^[^ ^20^ ^]^

## 4 Conclusions

Given the tumor‐agnostic characteristic of the GeM technology, it can be adopted for the treatment of many tumor types that are accessible via hypodermic needle or catheter. Furthermore, application of these materials can be extended to the concomitant local delivery of anticancer agents and immunomodulatory molecules. In summary, our innovative approach to mitigating tumor hypoxia has the potential to enhance the effectiveness of current cancer therapy.

## 5 Experimental Section

### Fabrication of GEMs

Foam GeM was fabricated similar to those described in the prior work.^[^ ^16^ ^]^ In brief, a prefoam solution composed of 1× phosphate buffered saline (PBS), xanthan gum (0.5 wt%), and methylcellulose (0.8 wt%) was generated through heating and stirring.^[^ ^15^ ^]^ The prefoam solution was degassed for >4 h after cooling, after which a foam was created by carefully inserting this solution into a modified iSi Pint whipping siphon with a custom‐made M22‐1/4 NPT connector (enables pressurization with a 99.5% oxygen cylinder). The whipping siphon was pressurized to 200 PSI with 99.5% oxygen (Linde). This foam GeM formulation was chosen to move forward with testing in vitro and in vivo due to the amount of gas entrapment and ease of use.

Hydrogel GeM was fabricated by creating an initial prefoam solution composed of sodium alginate (1.0 wt%), xanthan gum (0.25 wt%), and methylcellulose (0.8 wt%) in 1× PBS. This solution was degassed for >4 h after cooling. The hydrogels were then ionically cross‐linked in 100 mm calcium chloride solution.

Solid GeM was prepared using a Parr reactor (Series 4520 1L Bench Top Reactor, Parr Instrument Company, Moline, IL) with a 10 mL sample collection vessel attachment. A carbohydrate solution was generated by dissolving sucrose (42.6 wt%), lactose (42.6 wt%), and corn syrup (14.8 wt%) in deionized, distilled water. The solution was heated to 160 °C to achieve the appropriate water content, and it was then cooled to 135 °C. The heated solution was placed into the Parr reactor and stirred at 1000 rpm and pressurized to 600 PSI with 99.5% oxygen. It was stirred for 5 min and then allowed to flow into a heated sample collection vessel containing 0.8 mm (inner diameter) silicone tubing, where it was cooled to room temperature. The solid GeM was extracted from the tubing, cut to 5 mm lengths using a razor blade, and stored in desiccant at room temperature.

Various natural polymers were evaluated for their gas‐entrapping capacities as solid GeM. All solutions were handled as above. During qualitative evaluation of gas entrapment, the solid GeM was placed in room temperature water.

### Characterization of Materials

The materials were initially evaluated for bubble formation using an EVOS microscope at 4× magnification. Subsequently, oxygen gas in each GeM was quantified using a Hewlett Packard 6890 series gas chromatograph (GC) with a thermal conductivity detector. For evaluation of foam and hydrogel GeMs, GC vials were first subjected to multiple vacuum‐carbon dioxide purges and then the samples injected to the vials, in which they were shaken at 37 °C for 48 h. For evaluation of solid GeM, the samples were first placed into vials and then subjected to multiple vacuum‐carbon dioxide purges. In all cases, the samples were run in triplicate on the GC. Oxygen concentration was determined based upon calibration curves generated with 99.5% oxygen.

The foam and hydrogel GeMs additionally underwent rheological assessment using a TA Instruments DHR‐3 Rheometer at 37 °C. One percent strain was used for frequency sweeps, and amplitude sweeps were performed at 10 rad s^−1^. Studies of the volumetric stability of the foam were performed by transferring 200 mL foam to graduated cylinders and quantifying the volume and liquid fractions over time at 37 °C. Lastly, the solid natural polymer matrix of the solid GeMs was subjected to compressive testing to establish the fracture strength of the material. A 22 × 22 × 22 mm cube of the matrix was created and then subjected to compression under two parallel plates using an Instron machine at a rate of 10 mm min^−1^.

### Testing of Diffusion

The diffusion of oxygen from the foam GeM was evaluated in a tumor tissue surrogate (in 2 wt% agarose gel) using an Oxylite single channel dissolved oxygen (pO_2_) and temperature monitor system (Oxford Optronix, Oxford, UK). One milliliter of foam pressurized with 200 PSI of 99.5% O_2_ was deposited into a hole in the gel (7 mm in diameter). A 27‐gauge needle was used to introduce the oxygen sensor into the gel. Sensors were placed 1, 2, 3, 4, and 5 mm away from the site of foam deposition. The time to reach 20 mmHg above baseline was tested in 4–8 different samples per group.

### Testing of Cytotoxicity

The cytotoxicity of FluoroPel was evaluated by dissolving 20 mg of dried FluoroPel in Dulbecco's modified Eagle's medium (DMEM) with 10% fetal bovine serum (FBS) for 24 h. The medium was then filtered with a 0.2 µm filter and then diluted to various concentrations and applied to 5 × 10^3^ MyC‐CaP cells per well for 72 h. An alamarBlue assay was used to assess cell death^[^ ^21^ ^]^ in MPNST cells and to determine IC_50_ values for sorafenib and cabozantinib following exposure to room air and severely hypoxic environments (0.1% oxygen) in MyC‐CaP cells. These experiments were repeated four times.

### Animal Studies

C57BL/6NCrl (Strain Code: 027) and Balb/c mice were purchased from Charles River Laboratories. Mice were cared for in compliance with a protocol approved by the University of Iowa Institutional Animal Care and Use Committee (IACUC, #1092429‐012) and in adherence to the National Institutes of Health Guide for the Care and Use of Laboratory Animals.

As previously described, adenovirus containing Cas9 and sgRNAs targeting *Nf1* and *Cdkn2a* were made by the University of Iowa Viral Vector Core.^[^ ^22^ ^]^ Virus was mixed with calcium phosphate and DMEM and then tumors were generated by injecting 25 *µ*L of this mixture into the left sciatic nerve of a mouse. Mice were monitored for tumor development, and once a tumor reached a volume of at least 150 mm^3^ (Day 1), it was measured three times weekly using calipers. Tumor volume was approximated using the formula *V* = length (*L*) × width (*W*) × width (*W*)/2, with *L* and *W* corresponding to the *x* and *y* measurements of the tumor in mm. Animals were euthanized and tumors harvested once the volume of a tumor reached 1500 mm^3^.

Cell lines were derived from the terminally harvested MPNSTs as previously described.^[^ ^23^ ^]^ Briefly, at harvest, tumor tissue was finely minced with dissection scissors, washed with calcium‐ and magnesium‐deficient PBS, and then placed into a PBS‐based dissociation buffer containing collagenase type IV (Thermo) and dispase (Thermo) for 90 min at 37 °C on an orbital shaker. In a tissue culture hood, tissue was crushed in and passed through a 70 µm cell strainer (Fischer) with the removed plunger of a BD 1 mL syringe (Becton Dickson and Company), washed with calcium‐ and magnesium‐deficient PBS, and resuspended in DMEM (Gibco). Cells were subsequently cultured in DMEM containing 10% FBS, 1% penicillin–streptomycin (Gibco), and 1% sodium pyruvate (Gibco). Once cells demonstrated stable split patterns they were used for in vitro and in vivo experiments (beginning at ten passages).

Intramuscular (flank) MPNST tumors were generated by trypsinizing cultured MPNST cells, washing and resuspending them in PBS with calcium and magnesium, and then injecting the relevant muscle of C57BL/6NCrl mice with an optimized concentration of these cells using a 31‐guage needle. Specifically, gastrocnemius muscle was injected with 1.25 × 10^5^ cells in a 50 µL volume using a 31‐gauge needle. Subcutaneous MPNST tumors were generated by injecting the right subcutaneous flank with 3 × 10^5^ cells in a 50 µL volume. Prostate cancer tumors were generated by injecting the right subcutaneous flank C57BL/6NCrl mice with 3 × 10^6^ MyC‐CaP cells in 50 µL using a 31‐gauge needle.

To provide the most robust oxygen‐rich environment in and around the tumor, 50 µL of foam O_2_‐GeMs was intratumorally injected and 100 µL of foam O_2_‐GeMs was peri‐tumorally injected. The median size of MPNSTs at the time of GeM implantation was ≈160 mm^3^; that of subcutaneous syngeneic prostate tumors was ≈100 mm^3^. Tumors were measured using calipers two times weekly. Terminal tumor volume was set at 1500 mm^3^ for the MPNSTs and at 2000 mm^3^ for the MyC‐CaP tumors. All experiments used mice over 7 weeks old. All MPNST experiments used both male and female mice; prostate cancer studies used only male mice.

Intratumoral oxygen diffusion was measured using an Oxylite single channel dissolved oxygen (pO_2_) and temperature monitor system (Oxford Optronix, Oxford, UK). Isoflurane‐anesthetized C57BL6/J mice (Jackson Laboratory) were administered 50 µL foam pressurized with 200 PSI of 99.5% O_2_ via intratumoral injection 30 min, 1 h, 6 h, and 24 h before measurements were taken. An 18‐gauge angiocath was used to introduce the oxygen sensor into the tumor.

### CyTOF Analysis

Tumors were digested in a PBS‐based buffer (with 2% FBS) that contained both collagenase and DNAse. Subsequently, the cell homogenates were filtered through 70‐µm cell strainers (BD Biosciences) and immediately treated with CyTOF antibodies. For staining of cells, the cells were washed with 1× PBS, centrifuged at 300 × *g*, fixed with 1.6% formaldehyde, and then exposed to cisplatin viability staining reagent (Fluidigm) for ≈3 min. The cells were incubated in the antibody suspension for 30 min. The antibodies used for the CyTOF staining panel are listed in **Table** **2**. DNA was stained by adding an iridium intercalator solution (Fluidigm) to the cells. The cells were subsequently washed and reconstituted in Maxpar Cell Acquisition Solution with EQ four‐element calibration beads (Fluidigm) and then analyzed on a Helios CyTOF Mass Cytometer (Fluidigm).

**Table 2:** CyTOF antibodies used for staining

Marker	Metal
CD45	89Y
CD11b (Mac‐1)	148Nd
Ly‐6G/C (Gr‐1)	174Yb
F4/80	159Tb
CD86	172Yb
CD206 (MMR)	169Tm
CD3e	152Sm
CD4	145Nd
CD8a	168Er
CD62L (l‐selectin)	160Gd
CD44	171Yb
CD25 (IL‐2R)	150Nd
CD161 (NK1.1)	170Er
CD11c	209Bi
CD31 (PECAM‐1)	165Ho

CyTOF data were initially normalized using Normalizer and Debarcoding software. Data analysis was performed using the FlowJo v10 Software (BD Biosciences), Cytobank (Mountain View, CA), and the viSNE and CITRUS algorithms, enabling the mapping of single‐cell data in two dimensions as a tSNE plot. The tSNE plots were then used to understand the change in intratumoral microenvironment.

### Analysis of MHC Class I Expression

Flow cytometry was used to evaluate surface expression of MHC class I. Prior to staining, PBS with 1% FBS, 1% bovine serum albumin (BSA), 0.25 mm ethylenediaminetetraacetic acid (EDTA) was used to wash the samples, at 4 °C for 30 min. Samples were incubated with Alexa Fluor 488‐conjugated anti‐mouse H‐2Kb (clone AF6‐88.5; Biolegend) and PE‐Cy‐7‐conjugated anti‐mouse *β*2 microglobulin (clone A16041A; Biolegend) at 4 °C for 30 min. The cells were subsequently washed and resuspended in PBS with 1% FBS, 1% BSA, and 0.25 mm EDTA for acquisition of fluorescent signaling and size distribution on a BD LSR II cytometer custom configured for quality control beads at the Carver College of Medicine. Viability staining was accomplished using 7‐aminoactinomycin D (7‐AAD) exclusion (25 µg/1 × 106 cells) during acquisition. Apoptotic cells were detected by staining for annexin V‐APC (BD Pharmingen) according to the manufacturer's instructions. Control samples included single‐color staining, fluorescence‐minus‐one controls, and unstained samples. Spillover matrixes were calculated after single color compensation controls were acquired using the FlowJo Software V.10.5 (Becton, Dickinson and Company). Gate‐based analysis was used to exclude aggregates based on forward and side‐scatter characteristics, followed by viable cells based on Annexin‐ and 7AAD‐ cells. Assessed viability ranged from 94% to 96% across all experimental groups.

### Statistical Analyses

Data are expressed as mean ± standard deviation, except for flow data in Figure 6, which was presented as median ± standard error. Graphs were generated, and statistical analyses were performed, using GraphPad Prism Software V.6.01 for Windows (GraphPad Software, La Jolla, CA, USA). For comparisons of three or more groups, analysis of variance (ANOVA) methods were used. For comparisons of two groups, unpaired *t*‐tests were used and unadjusted *P* values are reported.

## Conflict of Interest

The authors declare no conflict of interest.

## Supporting information

Supporting Information

## Data Availability

The data that support the findings of this study are available from the corresponding author upon reasonable request.
