# Supplementary material for: Low‐Cost, High‐Pressure‐Synthesized Oxygen‐Entrapping Materials to Improve Treatment of Solid Tumors
Source: Adv Sci (Weinh). 2023 Feb 2;10(10):2205995. doi: 10.1002/advs.202205995 (PMC10074083; doi:10.1002/advs.202205995)
Supplement: Supplementary file 1 — Supporting Information [file ADVS-10-2205995-s001.pdf]

ADVANCED SCIENCE
Open Access

Supporting Information

for *Adv. Sci.*, DOI 10.1002/advs.202205995

Low-Cost, High-Pressure-Synthesized Oxygen-Entrapping Materials to Improve Treatment of Solid Tumors

*Jianling Bi, Emily Witt, Vanessa A. Voltarelli, Vivian R. Feig, Veena Venkatachalam, Hannah Boyce, Megan McGovern, Wade R. Gutierrez, Jeffrey D. Rytlewski, Kate R. Bowman, Ashley C. Rhodes, Austin N. Cook, Benjamin N. Muller, Matthew G. Smith, Alexis Rebecca Ramos, Heena Panchal, Rebecca D. Dodd, Michael D. Henry, Adam Mailloux, Giovanni Traverso, Leo E. Otterbein and James D. Byrne**

Supporting Information

Low-Cost, High-Pressure-Synthesized Oxygen-Entrapping Materials to Improve Treatment of Solid Tumors

*Jianling Bi, Emily Witt, Vanessa A. Voltarelli, Vivian R. Feig, Veena Venkatachalam, Hannah Boyce, Megan McGovern, Wade R. Gutierrez, Jeffrey D. Rytlewski, Kate R. Bowman, Ashley Rhodes, Austin Cook, Benjamin N. Muller, Mathew G. Smith, Alexis R. Ramos, Heena Panchal, Rebecca D. Dodd, Michael D. Henry, Adam Mailloux, Giovanni Traverso, Leo E. Otterbein, James D. Byrne**

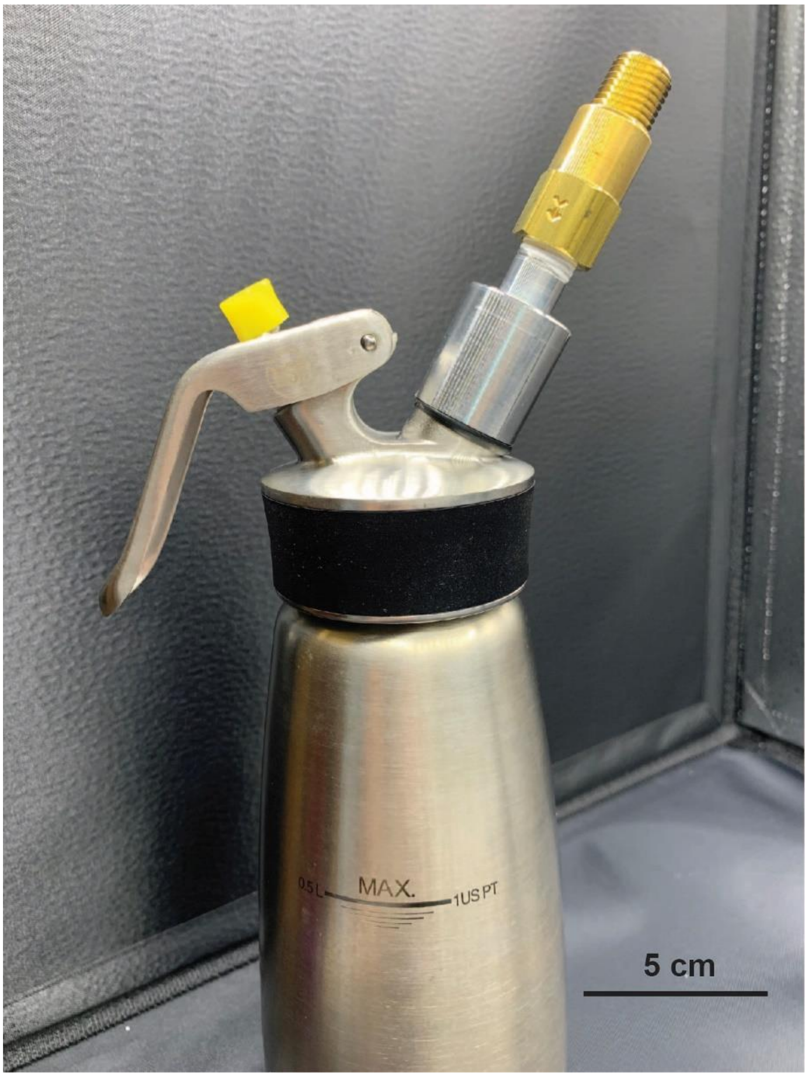

Figure S1: Modified whipping siphon for generating foam and hydrogel GeMs.

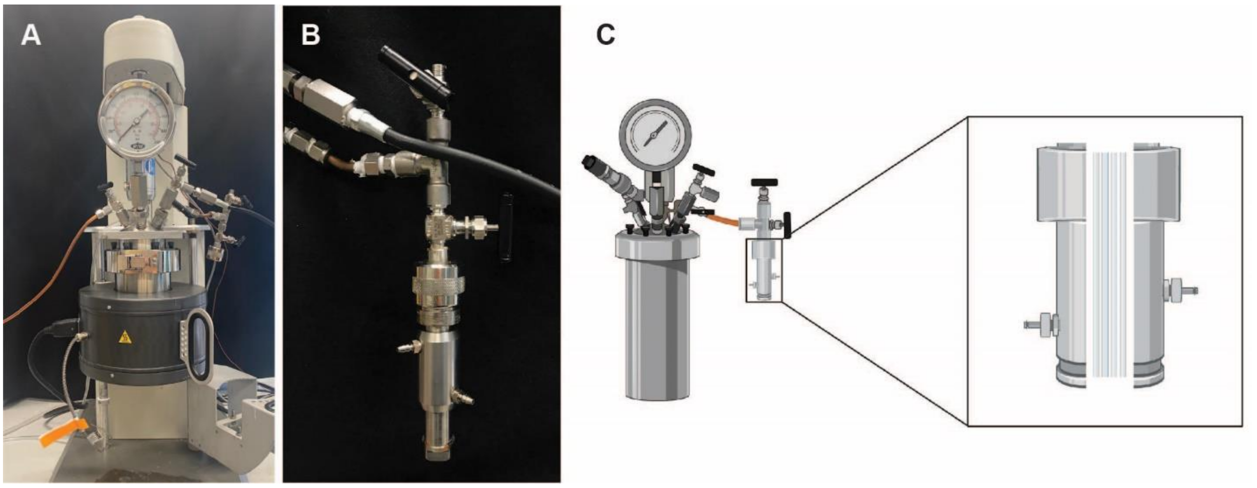

Figure S2: Parr reactor setup for producing solid GeMs. (A) Parr reactor, (B) sampling device, and (C) schematic of the sampling device, which makes it possible to generate clinically relevant solid GeMs that can be delivered through a catheter or hypodermic needle.

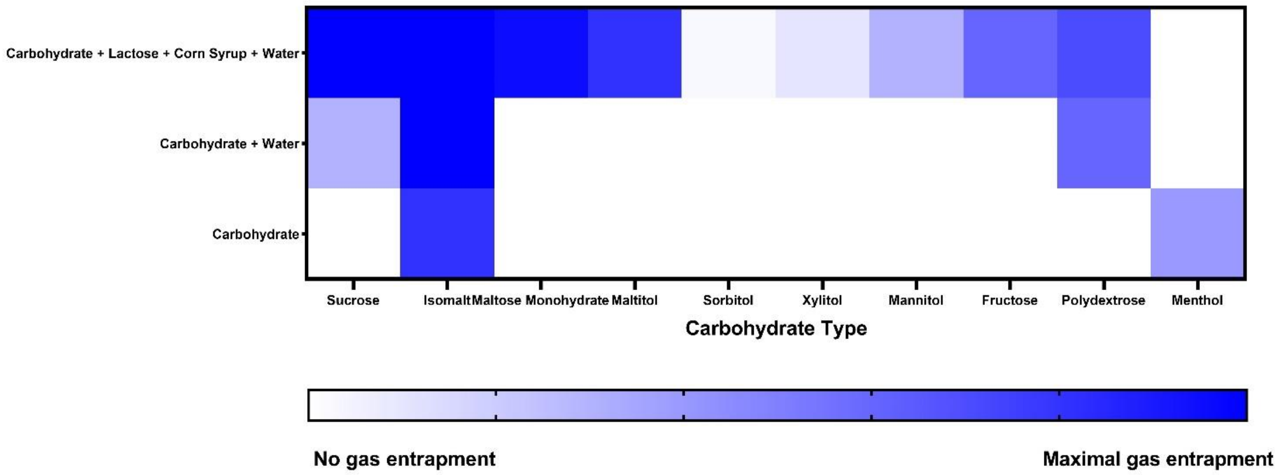

Figure S3: Qualitative assessment of gas entrapment from 10 different carbohydrate formulations. Each box shows the efficiency of gas entrapment based upon the carbohydrate formulation (x-axis).

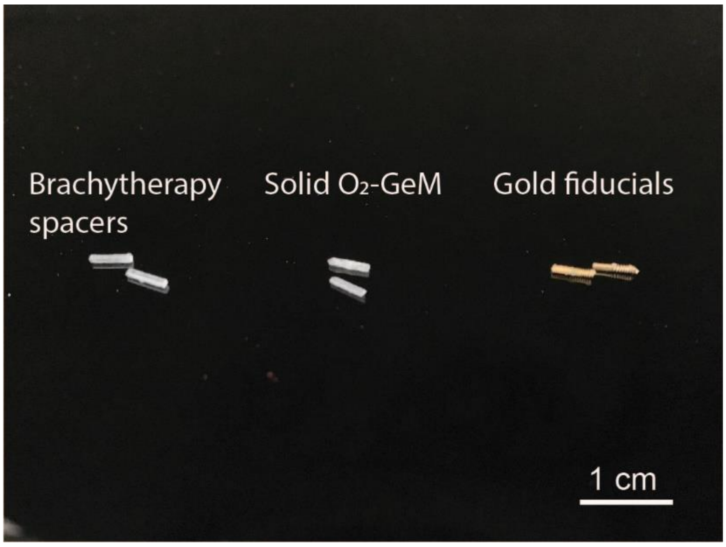

Figure S4: Comparison of size and shape of solid O_2-GeM to those of brachytherapy spacers and gold fiducials.

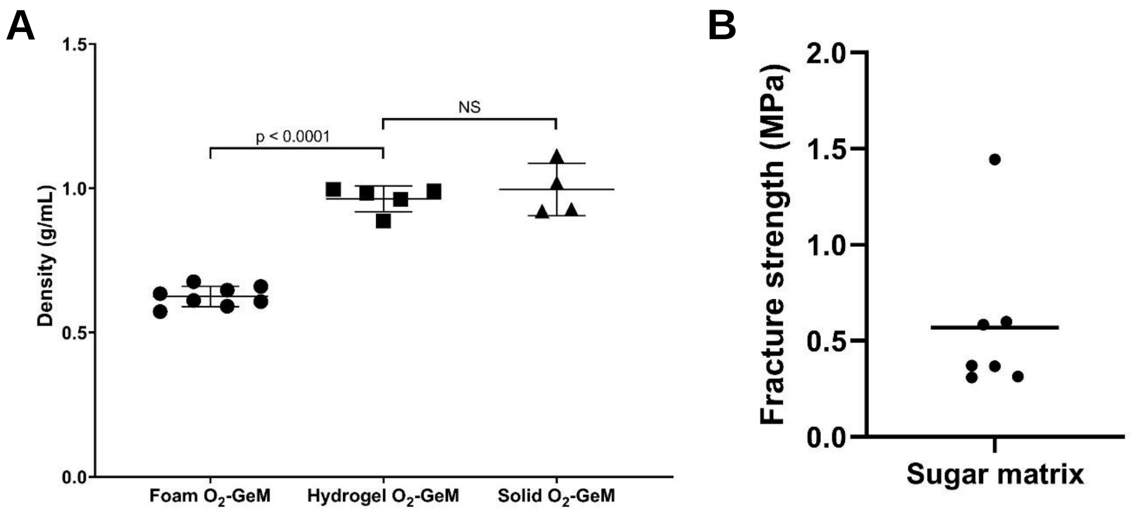

Figure S5: Material properties of density of each GeM type and the fracture strength of the natural polymer matrix used in solid O_2-GeMs. (A) Density of each O_2-GeM. (B) Fracture strength of natural polymer matrix measured using parallel plates on a mechanical tester. Data are means and standard deviations (n = 4-8 per arm). P values were determined by one-way ANOVA with multiple comparisons.

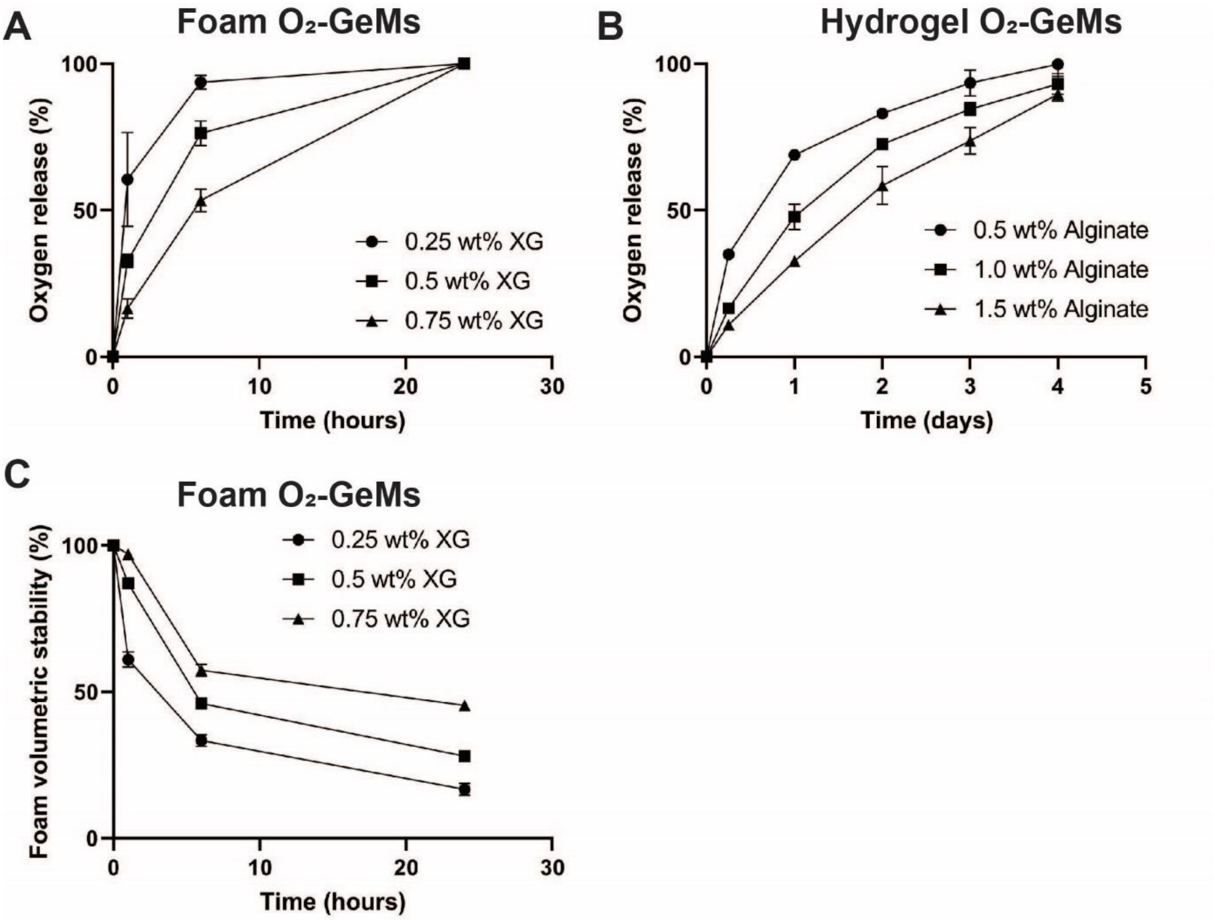

Figure S6: Material parameters of foam and hydrogel O_2-GeMs (A, B) Release kinetics of 100% O_2 from (A) foam O_2-GeM and (B) hydrogel O_2-GeM as a function of the concentrations of xanthan gum (XG) and alginate. (C) Volumetric stability of foam O_2-GeM as a function of the concentration of XG. Data are represented as means with standard deviations (n = 3 per group).

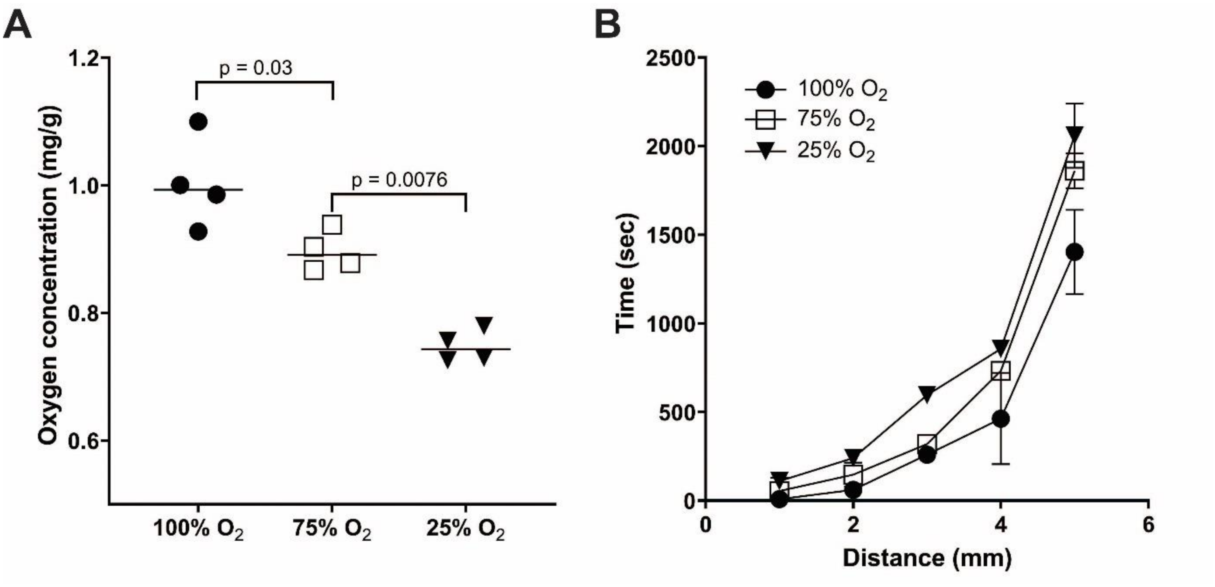

Figure S7: O_2 percentage in foam O_2-GeM influences gas delivery. (A) Oxygen concentration in the foam O_2-GeM as a function of the percentage of oxygen loaded into the whipping siphon, as measured using GC. (B) Oxygen diffusion within a tumor tissue surrogate (2 wt% agarose gel). The times recorded are those needed to reach 20 mmHg above baseline at oxygen probes implanted in the surrogate at the indicated distances. Data are represented as means with standard deviations (n = 4-8 per group). P values were determined by one-way ANOVA with multiple comparisons.

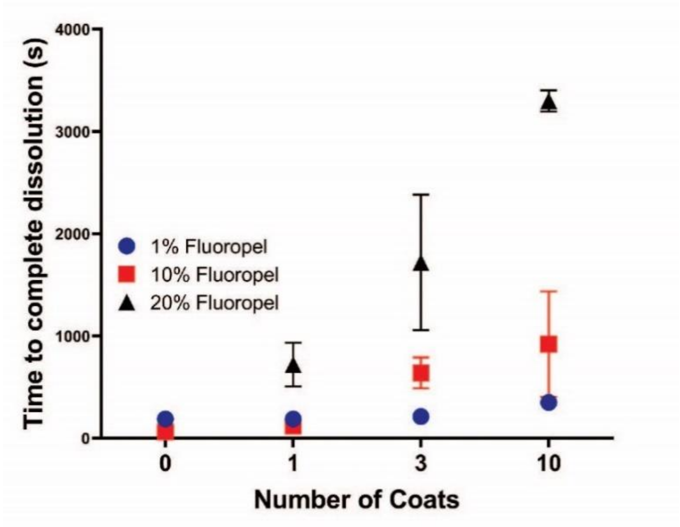

Figure S8: Impact of number of FluoroPel coats on rate of dissolution of the rapidly dissolvable matrix (n=5 per data point). Data are represented as means with standard deviation.

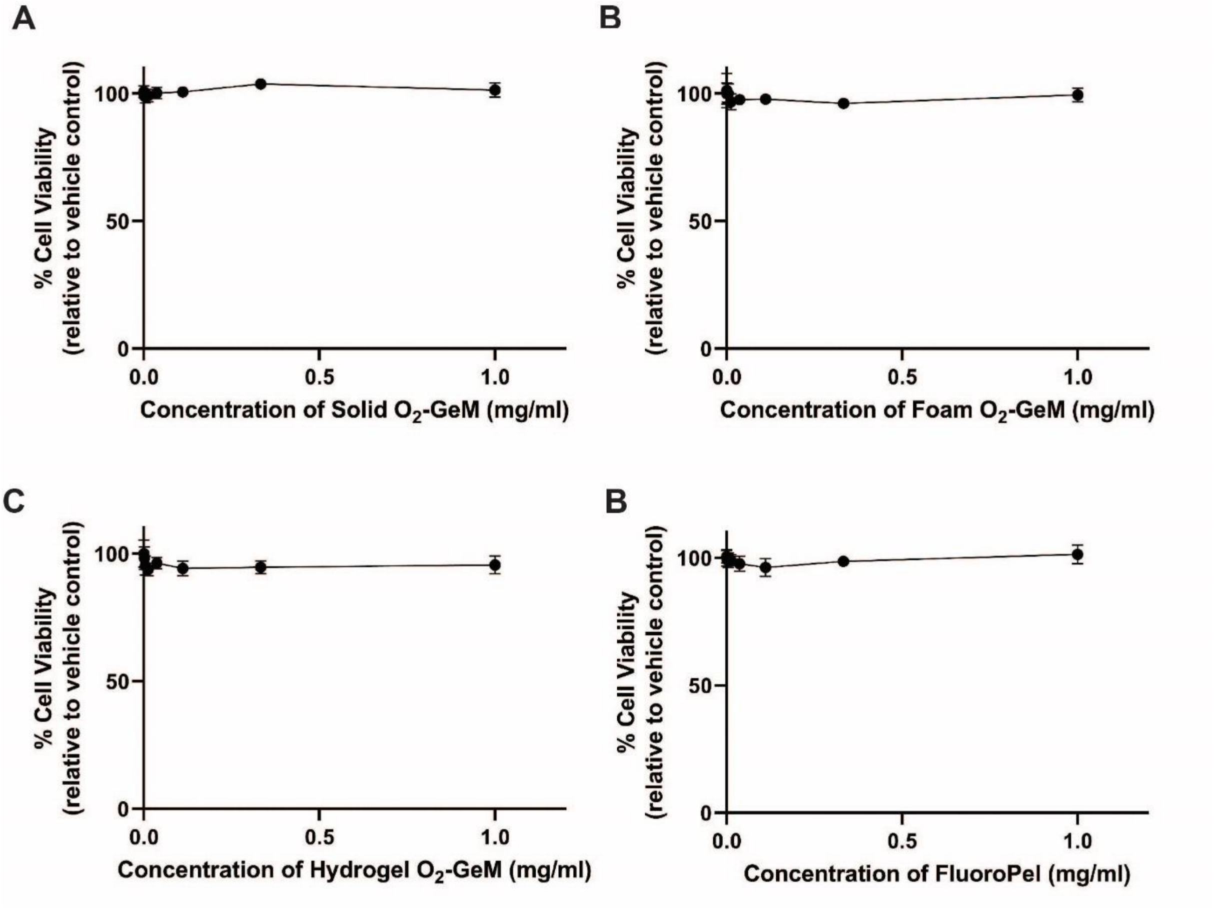

Figure S9: Evaluation of the biocompatibility of FluoroPel each GeM form tested. Cell viability in MyC-CaP cells as a function of the concentration of the (A) solid O_2-GeM, (B) foam O_2-GeM, or (C) hydrogel O_2-GeMs, or (D) FluoroPel coating. Cells were exposed to these materials for 72 hours and subsequently evaluated for cell viability using an alamarBlue assay (n = 8 wells/concentration). Data are represented as means with standard deviations.

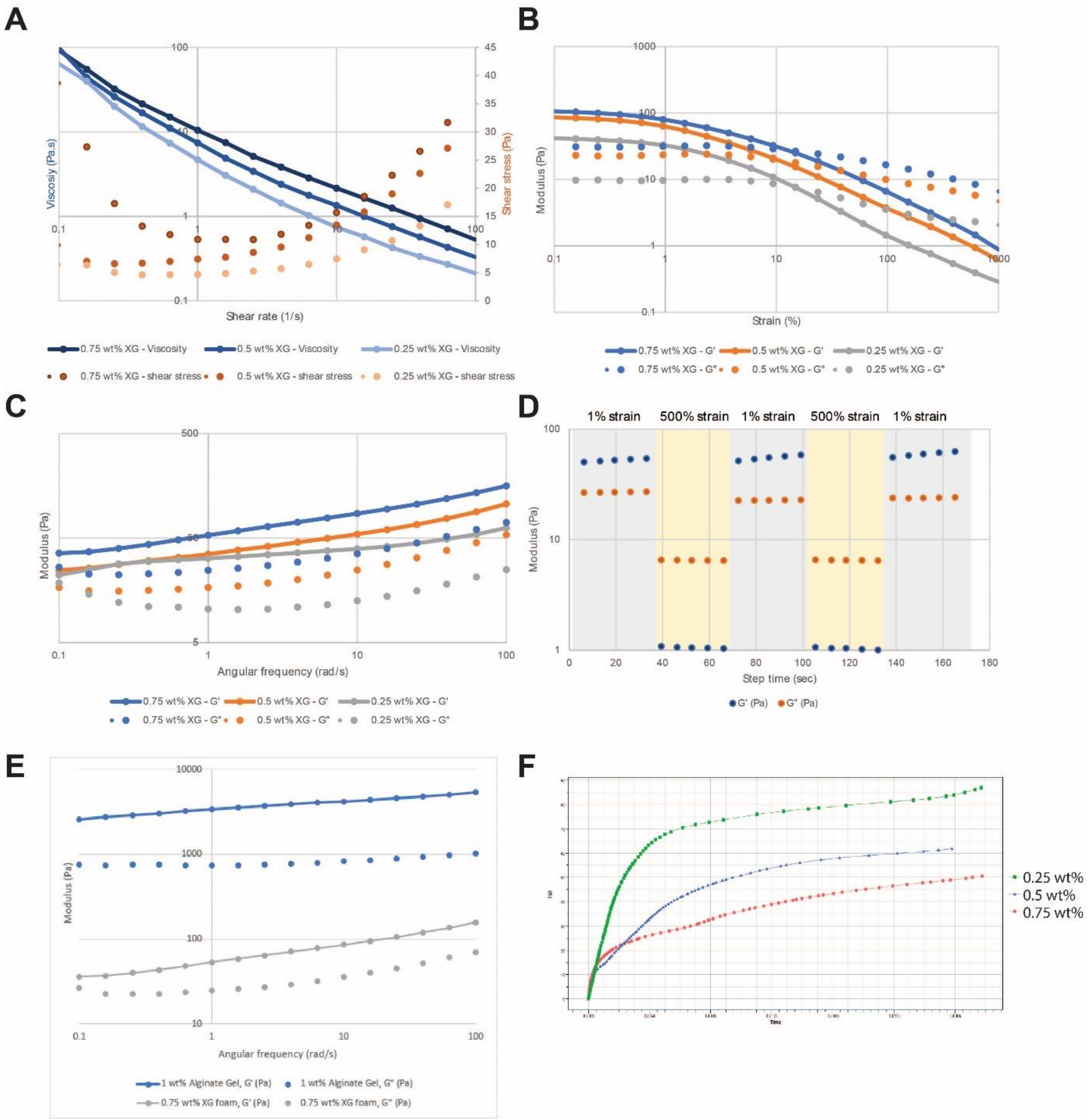

Figure S10: Rheologic assessment of foam O_2-GEMs. (A) Velocity and shear stress as functions of the shear rate, as assessed by oscillatory frequency sweeps at different concentrations of xanthan gum (XG) show that all formulations behave like viscoelastic solids (G’ > G”) and G’ increases with amount of xanthan gum (n = 3 per group). (B) Modulus of pressure as a function of the strain, which indicate that all formulations are highly shear-thinning (n = 3 per group). (C) Modulus of the pressure as a function of angular frequency that illustrate moderate shear strain values are sufficient to induce flow for all formulations (n = 3 per group). (D) Modulus of the pressure as a function of step time showing self-healing in foam GeM with 0.5 wt% XG foam, which immediately recovered elasticity (G’>G”) at 1%

strain after exposure to high shear strain of 500%. XG – xanthan gum (n = 3 per group). (E) Modulus of pressure as a function of angular frequency showing that hydrogel O_2-GeM at 1 wt% sodium alginate compared to foam O_2-GeM at 0.75 wt% xanthan gum has a higher degree of stiffness resulting in prolonged oxygen release. (F) Destabilization kinetics of the foams based upon the xanthan gum concentration (n = 3 per group).

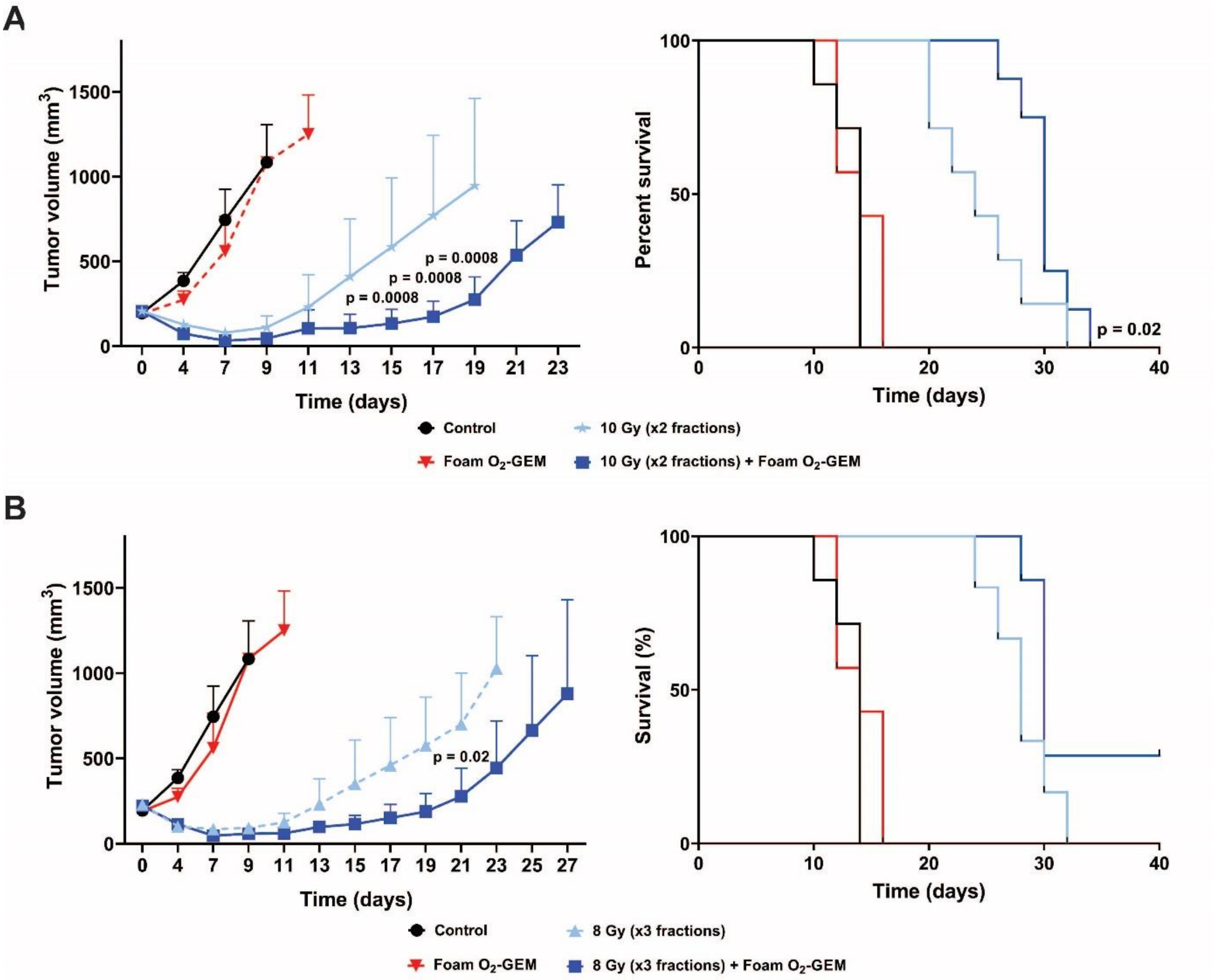

Figure S11: Effects of irradiation on tumor volume and percent survival in irradiated MPNST mice. Irradiation with (A) 10 Gy (x2 fractions) or (B) 8 Gy (x3 fractions). Both doses equivalent to the biologically effective dose (BED) used for pre-operative treatment of sarcoma tumors, assuming an alpha-beta of 4. Data are represented as means with standard deviations (n = 7 per arm).

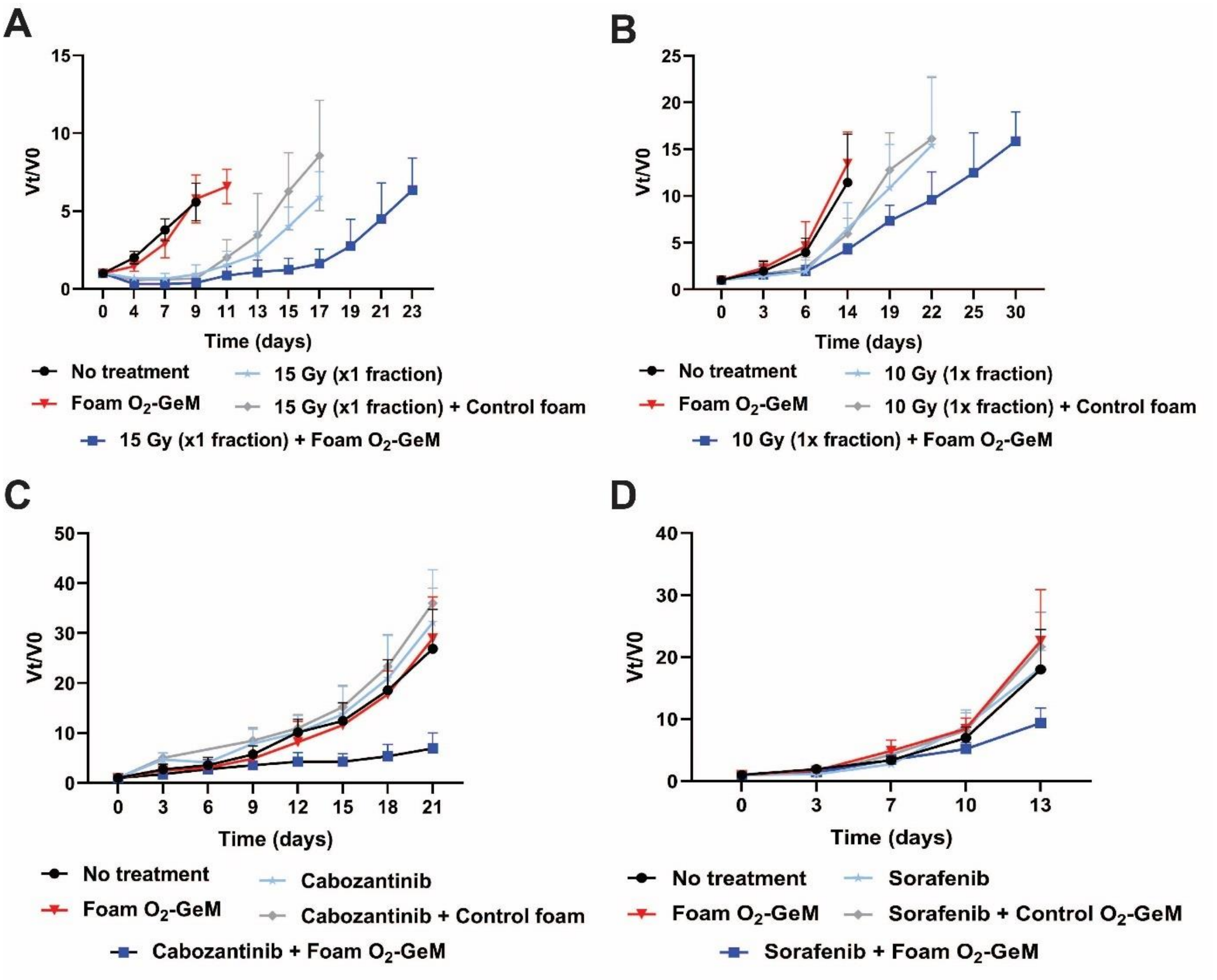

Figure S12: Efficacy of GeMs in inhibiting tumor growth. Growth curves plotted as $V_{t/}V_0$ for (A) intramuscular syngeneic MPNSTs implanted with foam O_2-GeM up to 30 minutes before irradiation (15 Gy); and (B) subcutaneous syngeneic prostate tumors injected with foam O_2-GeMs up to 30 minutes before irradiation (10 Gy); (C) foam O_2-GeMs intratumorally injected twice weekly with cabozantinib dosed in a subcutaneous syngeneic prostate cancer model; and (D) Sorafenib dosed in combination with intratumorally injected foam O_2-GeMs in an intramuscular (flank) syngeneic MPNST model. Data are represented as means with standard deviations (n = 7 per arm).

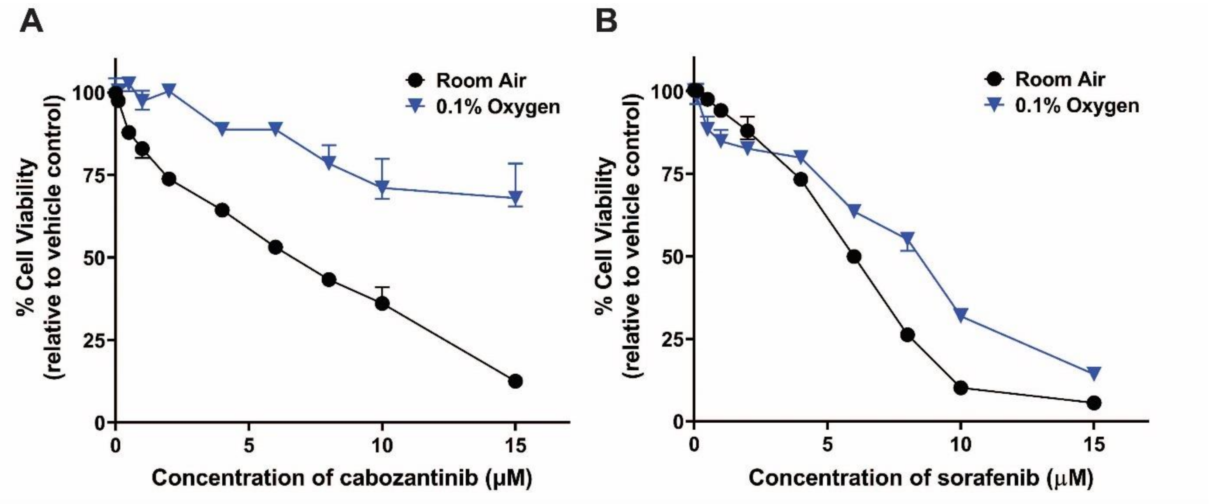

Figure S13: *In vitro* cytotoxicity of tyrosine kinase inhibitors in normoxia compared to hypoxia. IC_{50} values for (A) MyC-CaP cells treated with cabozantinib under normoxia (room air) or a severe hypoxia (0.1% oxygen) and (B) MPNST cells treated with sorafenib under the same conditions (n = 5 per concentration), as assessed by the alamarBlue viability assay at 48 hours after initial exposure. Data are represented as means with standard deviations.

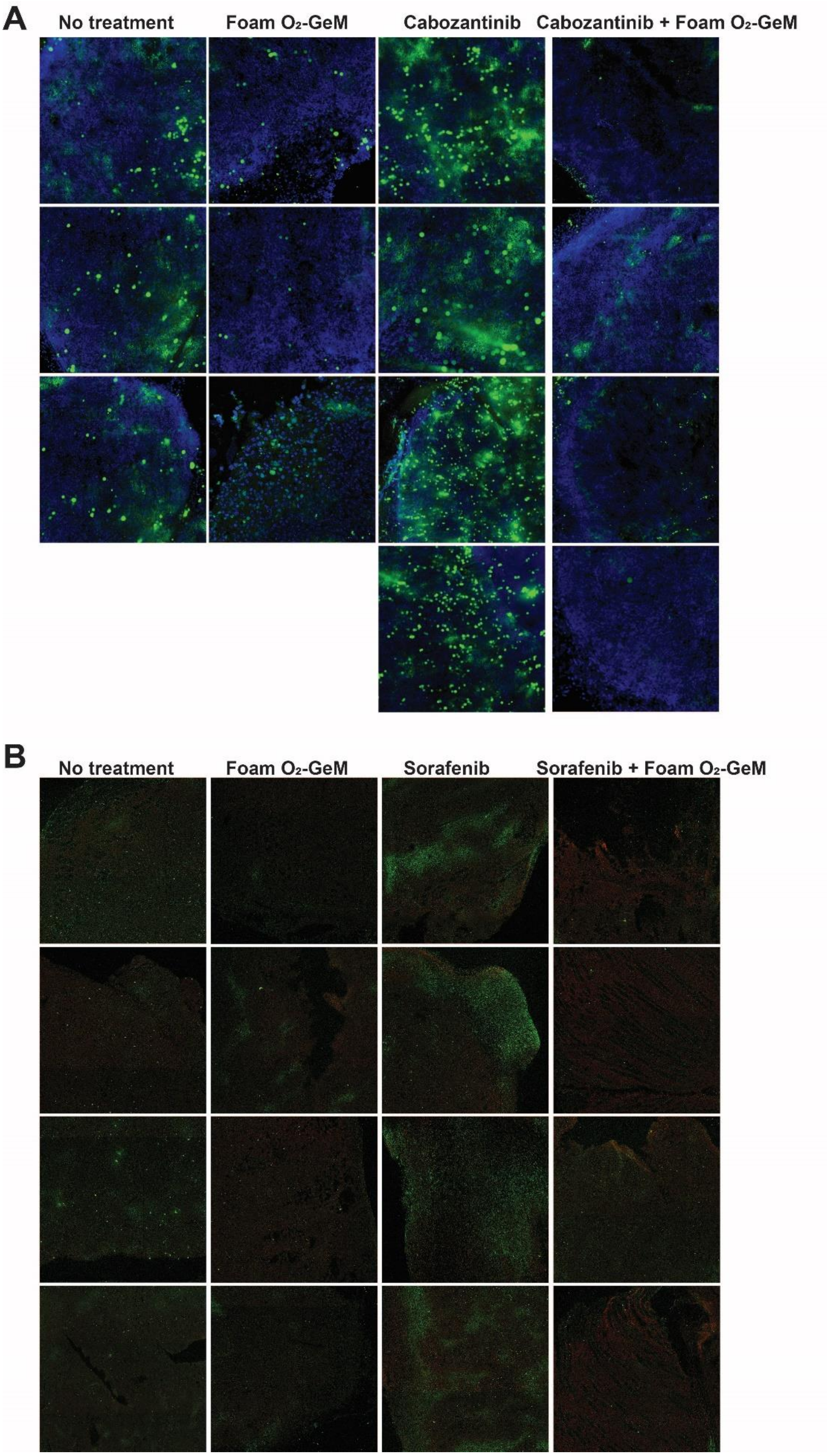

Figure S14. Histologic images of pimonidazole staining in (A) subcutaneous MyC-CaP tumors and (B) intramuscular MPNSTs 6 hours after treatment. Levels of pimonidazole staining from this figure and Figure 4I were quantified and shown in Figure 4H.
